# The Role of Angiotensin Converting Enzyme 1 Insertion/Deletion Genetic Polymorphism in the Risk and Severity of COVID-19 Infection

**DOI:** 10.3389/fmed.2021.798571

**Published:** 2021-12-23

**Authors:** Halim Saad, Karna Jabotian, Carine Sakr, Rami Mahfouz, Imad Bou Akl, Nathalie K. Zgheib

**Affiliations:** ^1^Department of Pharmacology and Toxicology, American University of Beirut Faculty of Medicine, Beirut, Lebanon; ^2^Employee Health Unit, Department of Family Medicine, American University of Beirut Faculty of Medicine, Beirut, Lebanon; ^3^Department of Pathology and Laboratory Medicine, American University of Beirut Faculty of Medicine, Beirut, Lebanon; ^4^Division of Pulmonary, Department of Internal Medicine, American University of Beirut Faculty of Medicine, Beirut, Lebanon

**Keywords:** *ACE1*, COVID, risk, severity, genetic polymorphism

## Abstract

**Background:** Individuals infected with the COVID-19 virus present with different symptoms of varying severity. In addition, not all individuals are infected despite exposure. Risk factors such as age, sex, and comorbidities play a major role in this variability; however, genetics may also be important in driving the differences in the incidence and prognosis of the disease. An *Insertion/Deletion* (*I/D*) polymorphism in the *ACE1* gene (rs1799752) may explain these genetic differences. The aims of this study were to determine the potential role of *ACE1 I/D* genetic polymorphism in the risk of contracting COVID-19 as well as predicting the severity of COVID-19 infection.

**Methods:** Three-hundred and eighty-seven non-related Lebanese subjects, 155 controls and 232 cases, who presented to the American University of Beirut Medical Center (AUBMC) for COVID-19 PCR testing were recruited. Clinical data were collected via filling a questionnaire and accessing the medical records. Peripheral blood was withdrawn for DNA isolation, and genotyping performed with standard PCR followed by band visualization on agarose gel.

**Results:** In our study population, previously described risk factors such as gender, age, and comorbidities were associated with increase in disease susceptibility and severity. *ACE1 I* was the least common allele, and there was a positive association between *ACE1 I* and the risk of contracting the COVID-19 disease. More specifically, the frequency of *II* genotype was significantly higher among cases when compared to controls (*P* = 0.035) with individuals with the *II* genotype having greater risk for contracting the COVID-19 disease: OR = 2.074, *P* = 0.048 in the multivariate analysis. As for disease severity, the *DD* genotype and *D* allele were associated with increased risk for developing severe symptoms (OR = 2.845, *P* = 0.026 and OR = 2.359, *P* = 0.014, respectively), and the *DD* genotype with necessitating hospitalization (OR = 2.307, *P* = 0.042). In parallel, *D* allele carriers showed a significantly increased risk for developing hypoxia: OR = 4.374, *P* = 0.045.

**Conclusion:** We found a positive association between *ACE1 I* and the risk of contracting the COVID-19 disease, and between *ACE1 D* and a worse outcome of the COVID-19 infection. Therefore, genotyping for *ACE1 I/D* polymorphism could be used to assess risk and predict severity for better prognosis and management of the disease.

## Introduction

Severe acute respiratory syndrome coronavirus 2 (SARS-CoV-2) is a positive-sense single-stranded RNA virus that is responsible for the globally transmissible coronavirus disease of 2019 (COVID-19) (1). It has been observed across infected populations worldwide that symptoms are displayed with dissimilar presentations of varying severity. In addition, not all individuals are infected despite a history of exposure, including multiple direct exposures, to COVID-19. Several factors have been described in the literature for their potential role in the risk of contracting COVID-19 as well as that of developing complications. These include age, sex, blood group, smoking history, comorbidities, obesity, and intake of ACE inhibitors (ACEI) or angiotensin receptor blockers (ARBs) (2–8). In addition to these risk factors, genetics may play a contributing role in COVID-19 infection (9). With inconclusive data, few studies have highlighted the roles of transmembrane protease serine 2 (*TMPRSS2*), angiotensin converting enzyme 1 (*ACE1*), and *ACE2* gene variants in the susceptibility and severity of SARS-CoV-2 infection (10–12).

TMPRSS2 expression facilitates the entry of the virus into host cells through *ACE2* (13). Both *ACE1* and *ACE2* are endogenous proteins involved in the renin-angiotensin system (RAS), which regulates the homeostasis of blood pressure and fluid electrolyte balance (14). In lung vascular endothelium, *ACE1* converts Angiotensin I into Angiotensin II that promotes vasoconstriction, inflammation, and thrombosis (14). *ACE2* converts Angiotensin II into Angiotensin 1–7 that acts inversely to Angiotensin II and hence promotes vasodilation (14). When SARS-CoV-2 enters human cells by binding its spike (S) protein to *ACE2*, lower levels of this membrane receptor become available for the suppression of Angiotensin II (15). Consequently, the balance of the RAS can be distorted in favor of vasoconstriction, inflammation, and thrombosis, potentially complicating the outcome of COVID-19 infection (14, 15).

An Insertion/Deletion (*I/D*) polymorphism in the *ACE1* gene (rs1799752) may explain the differences in genetic susceptibilities across variable geographic populations. The *ACE1 D/D* genotype correlates with a higher activity of the *ACE1* enzyme, hence increasing the levels of Angiotensin II with secondary lowering of *ACE2* expression (16). Despite some negative results (17), few studies showed the *DD* genotype to be associated with a significantly higher risk for COVID-19 morbidity and mortality (18, 19). Moreover, a higher *I/D*-allele frequency ratio has been associated with higher recovery rates despite an increase in infectivity (20). A comprehensive review done in 2021 regarding the association between *ACE1* (*I/D*) polymorphism and COVID-19 symptoms is referenced for the reader (21). The data are less conclusive concerning the association between *ACE1* (*I/D*) genetic polymorphism and risk of contracting the disease. For instance, an initial analysis by Delanghe et al. (22) of disease spread in 25 European countries with *ACE1* historical genetic data showed a significant association between COVID-19 cases and higher frequency of the *ACE1 I* allele (22). In contrast, Yamamoto et al. (23) observed that the Europeans have a higher probability of being infected by SARS-CoV-2 compared to Asian populations who have a higher frequency of the *ACE1 II* genotype. Importantly, the negative correlation between COVID-19 incidence and *ACE1 II* genotype was weakened when they added data from the Middle East, stating that the Middle East should be considered an important factor for future studies (23). This is especially the case since, and as per Saab et al. (24), the Middle Eastern population such as the Lebanese, have a lower frequency of the *ACE1 I* allele when compared to the *D* allele.

The aims of this study were to determine the potential role of *ACE1 I/D* genetic polymorphism in the risk of contracting COVID-19 as well as predicting the severity of COVID-19 infection. We hypothesized that the *ACE1 I* allele is associated with an increased risk of contracting the SARS-CoV-2 virus, while the *ACE1 D* allele is associated with a worse prognosis depicted as increased severity of signs, symptoms, and sequelae following COVID-19 infection.

## Methods

### Human Subjects

This study was approved by the Institutional Review Board (IRB) of the American University of Beirut (AUB). Three-hundred and eighty-seven Lebanese adult subjects were recruited given they had presented to the AUB Medical Center (AUBMC) for COVID-19 PCR testing (irrespective of result), COVID-19 hospitalization, or post-COVID-19 persistent symptoms. The recruitment process entailed a one-time participation that included informed consent process, data collection, and peripheral blood withdrawal for DNA isolation and *ACE1* genotyping.

### Data Collection

Data for this study were obtained via a questionnaire and access through medical records on the electronic heath information system EPIC. Information collected included demographics, comorbidities, medications intake, date of PCR testing, and COVID-19 disease presentation, management, and progression for each participant.

### ACE1 I/D Genotyping

Peripheral blood was collected in EDTA containing tubes, processed into aliquots and stored at −80°C. DNA was then isolated using FlexiGene^®^ DNA Kit by QIAGEN^®^ (Germany) as per the manufacturer's guidelines. Isolated DNA was read using the DS-11 Spectrophotometer (DeNovix^®^, USA) for quantification and purity assessment and stored at −20°C. Genotyping for *ACE1 insertion/deletion* polymorphism was carried out by polymerase chain reaction (PCR) followed by gel visualization with primers and experimental conditions as previously described (25). Individuals homozygous for the *D* allele and *I* allele were identified by a single 190 bp fragment and a single 490 bp fragment, respectively. Heterozygous individuals were identified by the presence of both fragments.

### Statistical Analysis

The collected data were transcribed onto Microsoft Excel then exported to SPSS^®^ (IBM, USA) for description and analysis. A *P* < 0.05 was considered statistically significant.

The *ACE1* polymorphism was analyzed using four separate associations: one for the alleles (*I* vs. *D*), and the remaining three for the genotypes (*II* vs. *DI* vs. *DD, D*-carriers, and *I*-carriers). The *D*-carrier association was (*II* vs. *DI* + *DD*), and that of the *I*-carrier was (*DD* vs. *DI* + *II*). The genotype frequencies in controls were checked for Hardy Weinberg Equilibrium (HWE) using chi-square test.

Baseline characteristics included in the analysis were age, body mass index (BMI), sex, blood group (containing *A* or not), smoking (never, ever), comorbidities, and intake of ACEI or ARBs. Comorbidities were classified as follows: dyslipidemia, hypertension, diabetes, heart disease (coronary artery disease or heart failure), kidney disease (chronic kidney disease or end-stage renal disease), lung disease (chronic obstructive pulmonary disease or interstitial lung disease or asthma), cerebrovascular disease (stroke or carotid stenosis), coagulation disorders (hemophilia or von Willebrand disease), and cancer.

For the association of *ACE1* (*I/D*) polymorphism with contracting COVID-19 disease, participants infected with COVID-19 (cases) were compared to those who were not (controls). For the association of *ACE1* (*I/D*) polymorphism with severity and outcome of COVID-19 infection, three comparisons were carried out: mild vs. moderate vs. severe disease, hospitalized vs. non-hospitalized, and hypoxic (SpO_2_ <94%) vs. non-hypoxic (SpO_2_ ≥94%) upon hospitalization. Disease severity was classified according to the WHO clinical progression scale into three stages: stage I (mild), stage II (moderate), and stage III (severe) (26). Mild presentation included any combination of the following: fever and/or chills, cough, shortness of breath, sore throat, congestion and/or rhinorrhea, fatigue, myalgias, headache, nausea and/or vomiting, diarrhea, anosmia, and ageusia. The moderate disease stage included symptomatic patients who were hospitalized with evident radiographic lung inflammation and a blood oxygen saturation (SpO_2_) ≥94% with minimal or no oxygen therapy required (26). Severe disease included critically ill patients with marked lung infiltrates on Chest X-Ray or CT scan and hypoxia (SpO_2_ <94%) who required hospitalization with essential oxygen therapy by either nasal cannula, face mask, non-invasive ventilation (NIV), and/or mechanical ventilation with intubation (26).

Association analyses were carried out using Fisher's Exact test for categorical variables and independent sample *t*-test or one-way ANOVA with *post-hoc* Bonferroni for continuous variables as applicable. Binary or multinomial logistic regressions were used for the associations with *ACE1* (*I/D*) polymorphism at both the univariate and multivariate level since these are the main focus of the study. Multivariate regression entailed adjustment for all statistically significant covariates at the univariate level. Results are presented as number (percentage) *N* (%), mean ± standard deviation (SD) at the univariate level, and odds ratios (OR) (adjusted and unadjusted) with 95% confidence intervals.

Additional analysis was performed to explore previously reported association(s) of the *ACE1* polymorphism with comorbidities.

## Results

Three-hundred and eighty-seven non-related Lebanese subjects, 155 controls and 232 cases, who presented to AUBMC for COVID-19 PCR testing were recruited and included in this study. The three genotypes were in HWE (*P* = 0.281). *ACE1 I* allele was the least common with a frequency of 31.0% and a *II* genotype frequency of 7.8% in controls (Table 1). These numbers are in line with the literature stating that the *I* allele is least common in Caucasians and Middle Easterners, and most common in Asians [Supplementary Table 1; (24, 27)].

**Table 1:** Association between baseline characteristics and *ACE1* polymorphism in COVID-19 positive cases vs. COVID-19 negative controls.

			**Controls** ***N*** **= 155**	**Cases** ***N*** **= 232**	***P*** **-Value^a^**
Age (years)	Mean ± SD		37.14 ± 11.48	43.75 ± 15.85	**<0.001**
BMI^b^ (kg/m^2^)	Mean ± SD		25.79 ± 4.14	27.82 ± 5.51	**<0.001**
Sex	Female	*N* (%)	86 (55.5)	106 (45.7)	**0.037**
	Male	*N* (%)	69 (44.5)	126 (54.3)	
Blood group A+	Yes	*N* (%)	75 (48.4)	118 (50.9)	0.354
	No	*N* (%)	80 (51.6)	114 (49.1)	
Smoking	Ever	*N* (%)	63 (40.6)	98 (42.2)	0.418
	Never	*N* (%)	92 (59.4)	134 (57.8)	
Dyslipidemia	Yes	*N* (%)	19 (12.3)	40 (17.2)	0.116
	No	*N* (%)	136 (87.7)	192 (82.8)	
Hypertension	Yes	*N* (%)	11 (7.1)	46 (19.8)	**<0.001**
	No	*N* (%)	144 (92.9)	186 (80.2)	
Diabetes	Yes	*N* (%)	4 (2.6)	29 (12.5)	**<0.001**
	No	*N* (%)	151 (97.4)	203 (87.5)	
Heart disease^c^	Yes	*N* (%)	2 (1.3)	15 (6.5)	**<0.001**
	No	*N* (%)	153 (98.7)	217 (93.5)	
Kidney disease^d^	Yes	*N* (%)	1 (0.6)	8 (3.4)	0.068
	No	*N* (%)	154 (99.4)	224 (96.6)	
Lung disease^e^	Yes	*N* (%)	5 (3.2)	13 (5.6)	0.202
	No	*N* (%)	150 (96.8)	219 (94.4)	
Cerebrovascular disease^f^	Yes	*N* (%)	0 (0.0)	2 (0.9)	0.359
	No	*N* (%)	155 (100)	230 (99.1)	
Coagulation disorders^g^	Yes	*N* (%)	1 (0.6)	4 (1.7)	0.335
	No	*N* (%)	154 (99.4)	228 (98.3)	
Cancer	Yes	*N* (%)	3 (1.9)	21 (9.1)	**0.003**
	No	*N* (%)	152 (98.1)	211 (90.9)	
ACEI^h^/ARB^i^ intake	Yes	*N* (%)	9 (5.8)	27 (11.6)	**0.037**
	No	*N* (%)	146 (94.2)	205 (88.4)	
*ACE* genotype	*II*	*N* (%)	12 (7.8)	33 (14.2)	0.141
	*DI*	*N* (%)	72 (46.4)	104 (44.8)	
	*DD*	*N* (%)	71 (45.8)	95 (41.0)	
	*II*	*N* (%)	12 (7.8)	33 (14.2)	**0.035**
	*DI* + *DD*	*N* (%)	143 (92.3)	199 (85.8)	
	*DI* + *II*	*N* (%)	84 (54.2)	137 (59.1)	0.200
	*DD*	*N* (%)	71 (45.8)	95 (41.0)	
*ACE* allele	*I*	*N* (%)	96 (31.0)	170 (36.6)	0.060
	*D*	*N* (%)	71 (69.0)	95 (63.4)	

a *P-values defined using independent t-test for continuous variables and Fisher exact for categorical variables*.

b *Body mass index*.

c *Coronary artery disease; heart failure*.

d *Chronic kidney disease, end-stage renal disease*.

e *Chronic obstructive pulmonary disease, interstitial lung disease, asthma*.

f *Stroke, carotid stenosis*.

g *Hemophilia, von Willebrand disease*.

h *Angiotensin converting enzyme inhibitor*.

i *Angiotensin receptor blocker*.

*The statistically significant P values are in bold*.

### Disease Susceptibility

When comparing baseline characteristics to predict disease susceptibility in cases vs. controls (Table 1), the cases were both older and of higher BMI. There was a larger proportion of males in the infected group compared to that of the uninfected group. Hypertension, diabetes, heart disease, and cancer were all significant comorbid predictors for COVID-19 susceptibility. Moreover, there was a greater proportion of participants taking ACEI/ARB_s_ among the case group when compared to controls (Table 1).

**Figure 1:**
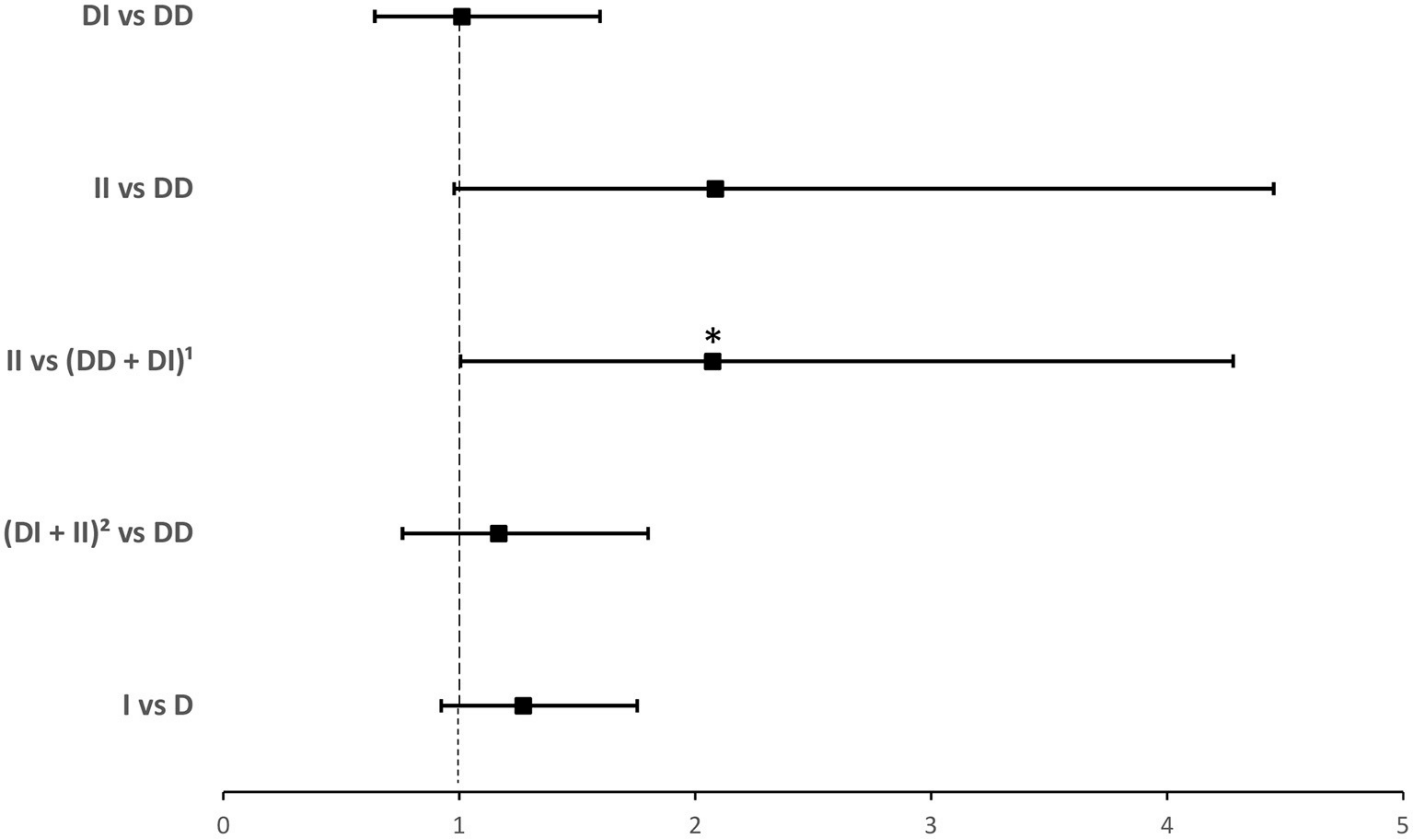
Forest plot showing adjusted odds ratios ± 95% confidence intervals of *ACE1* polymorphism for contracting SARS-CoV-2. Multivariate analysis included variables that were statistically significant in the association analysis shown in Table 1; *P < 0.05. ^1^*D* allele carriers. ^2^*I* allele carriers.

Compared to *ACE1 D*, the frequency of the *II* genotype was significantly higher among individuals infected with COVID-19 (14.2 vs. 7.8%; *P* = 0.035; Table 1). After adjusting for age, BMI, sex, hypertension, diabetes, heart disease, cancer, and ACEI/ARB_s_ intake, binary logistic regression showed that, compared to *D* allele carriers, individuals with the *II* genotype were at increased risk for contracting the virus (OR = 2.074; *P* = 0.048; Supplementary Table 2 and Figure 1).

### Disease Severity

Among the 232 cases, 223 were symptomatic: 136 (61.0%) had mild symptoms, 26 (11.7%) had moderate symptoms and 61 (27.3%) had severe symptoms. The mean ± SD of symptoms' duration was 10.14 ± 8.56 days.

As show in Table 2, compared to cases with mild infection, those with moderate and severe infection were older and of higher BMI. There were larger proportions of males among moderate and severe cases compared to mild cases. Dyslipidemia, hypertension, diabetes, heart disease, kidney disease, coagulation disorders, and cancer were significant comorbid predictors for moderate and severe disease vs. mild disease. There was also a larger proportion of ACEI/ARB_s_ intake among moderate and severe cases when compared to controls (Table 2).

**Table 2:** Association between baseline characteristics and *ACE1* polymorphism with disease severity^1^ in symptomatic COVID-19 cases.

			**Mild** ***N*** **= 138**	**Moderate** ***N*** **= 26**	**Severe** ***N*** **= 61**	***P*** **-Value^2^**
Age (years)	Mean ± SD		36.51 ± 11.06^a^, ^b^	54.00 ± 15.03	56.98 ± 15.33	**<0.001**
BMI^3^ (kg/m^2^)	Mean ± SD		26.55 ± 4.87^b^	27.85 ± 4.56^c^	31.05 ± 6.18	**<0.001**
Sex	Female	*N* (%)	75 (55.1)	7 (26.9)	18 (29.5)	**0.001**
	Male	*N* (%)	61 (44.9)	19 (73.1)	43 (70.5)	
Blood group A+	Yes	*N* (%)	70 (51.5)	11 (42.3)	32 (52.5)	0.682
	No	*N* (%)	66 (48.5)	15 (57.7)	29 (47.5)	
Smoking	Ever	*N* (%)	57 (41.9)	11 (42.3)	25 (41.0)	1.000
	Never	*N* (%)	79 (58.1)	15 (57.7)	36 (59.0)	
Dyslipidemia	Yes	*N* (%)	14 (10.3)	6 (23.1)	20 (32.8)	**0.001**
	No	*N* (%)	122 (89.7)	20 (76.9)	41 (67.2)	
Hypertension	Yes	*N* (%)	10 (7.4)	8 (30.8)	28 (45.9)	**<0.001**
	No	*N* (%)	126 (92.6)	18 (69.2)	33 (54.1)	
Diabetes	Yes	*N* (%)	5 (3.7)	6 (23.1)	18 (29.5)	**<0.001**
	No	*N* (%)	131 (96.3)	20 (76.9)	43 (70.5)	
Heart disease^4^	Yes	*N* (%)	1 (0.7)	4 (15.4)	10 (16.4)	**<0.001**
	No	*N* (%)	135 (99.3)	22 (84.6)	51 (83.6)	
Kidney disease^5^	Yes	*N* (%)	1 (0.7)	3 (11.5)	4 (6.6)	**0.003**
	No	*N* (%)	135 (99.3)	23 (88.5)	57 (93.4)	
Lung disease^6^	Yes	*N* (%)	7 (5.1)	3 (11.5)	3 (4.9)	0.367
	No	*N* (%)	129 (94.9)	23 (88.5)	58 (95.1)	
Cerebrovascular disease^7^	Yes	*N* (%)	0 (0.0)	1 (3.8)	1 (1.6)	0.077
	No	*N* (%)	136 (100.0)	25 (96.2)	60 (98.4)	
Coagulation disorders^8^	Yes	*N* (%)	0 (0.0)	0 (0.0)	4 (6.6)	**0.010**
	No	*N* (%)	136 (100.0)	26 (100.0)	57 (93.4)	
Cancer	Yes	*N* (%)	1 (0.7)	9 (34.6)	11 (18.0)	<**0.001**
	No	*N* (%)	135 (99.3)	17 (65.4)	50 (82.0)	
ACEI^9^/ARB^10^ intake	Yes	*N* (%)	9 (6.6)	4 (15.4)	14 (23.0)	**0.005**
	No	*N* (%)	127 (93.4)	22 (86.4)	47 (77.0)	
*ACE* genotype	*II*	*N* (%)	23 (16.9)	5 (19.2)	5 (8.2)	0.348
	*DI*	*N* (%)	62 (45.6)	12 (46.2)	26 (42.6)	
	*DD*	*N* (%)	51 (37.5)	9 (34.6)	30 (49.2)	
	*II*	*N* (%)	23 (16.9)	5 (19.2)	5 (8.2)	0.200
	*DI* + *DD*	*N* (%)	113 (83.1)	21 (80.8)	56 (91.8)	
	*DI* + *II*	*N* (%)	85 (62.5)	17 (65.4)	31 (50.8)	0.259
	*DD*	*N* (%)	51 (37.5)	9 (34.6)	30 (49.2)	
*ACE* allele	*I*	*N* (%)	108 (39.7)	22 (42.3)	36 (29.5)	0.15
	*D*	*N* (%)	164 (60.3)	30 (57.7)	86 (70.5)	

1 *Rated as mild, moderate, or severe according to the WHO clinical progression scale for COVID-19*.

2 *P-values defined using one-way ANOVA with post-hoc Bonferroni for continuous variables and Fisher exact for categorical variables*.

a *P < 0.05 for Mild vs. Moderate with post-hoc Bonferroni*.

b *P < 0.05 for Mild vs. Severe with post-hoc Bonferroni*.

c *P < 0.05 for Moderate vs. Severe with post-hoc Bonferroni*.

3 *Body mass index*.

4 *Coronary artery disease; heart failure*.

5 *Chronic kidney disease, end-stage renal disease*.

6 *Chronic obstructive pulmonary disease, interstitial lung disease, asthma*.

7 *Stroke, carotid stenosis*.

8 *Hemophilia, von Willebrand disease*.

9 *Angiotensin converting enzyme inhibitor*.

10 *Angiotensin receptor blocker*.

*The statistically significant P values are in bold*.

**Figure 2:**
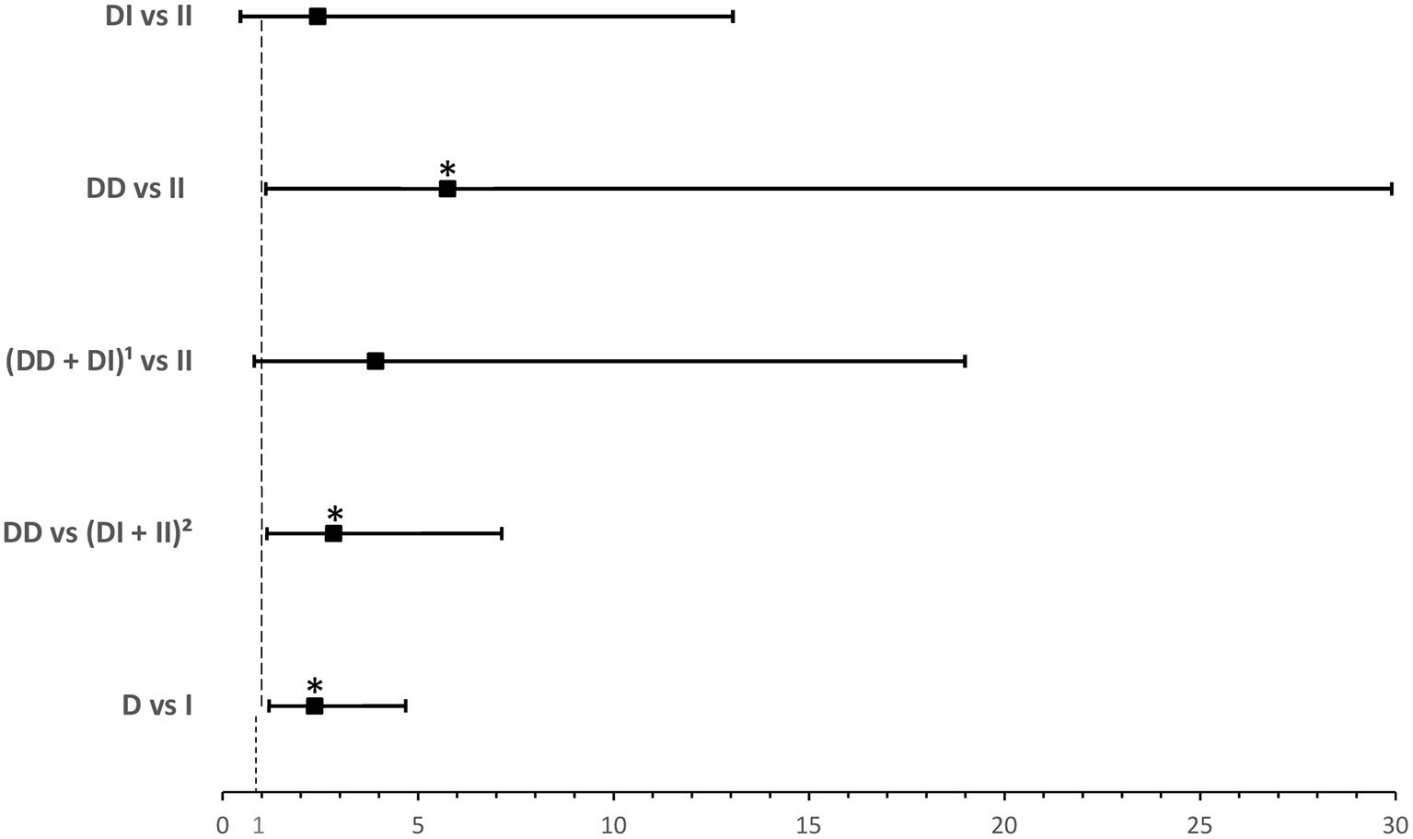
Forest plot showing odds ratios ± 95% confidence intervals of *ACE1* polymorphism for developing severe^a^ disease in symptomatic COVID-19 cases. Multivariate analysis included variables that were statistically significant in the association analysis shown in Table 2; ^*^P < 0.05. ^a^Rated as mild, moderate, or severe according to WHO clinical progression scale for COVID-19 with mild disease as Reference. ^1^*D* allele carriers. ^2^*I* allele carriers.

*ACE1 I/D* genotype and allele frequencies were not significantly associated with disease severity although there was a trend of higher *DD* genotype and *D* allele frequencies in cases with severe symptoms of COVID-19 disease (Table 2). After adjusting for age, BMI, sex, significant comorbidities, and ACEI/ARB_s_ intake, multinomial logistic regression showed that symptomatic cases with the *DD* genotype had a higher risk of developing severe disease following SARS-CoV-2 infection (OR = 5.751; *P* = 0.038) when compared to symptomatic *II* individuals. In addition, and compared to symptomatic *I* carriers, symptomatic cases with the *DD* genotype were more likely to develop severe disease following infection (OR = 2.845; *P* = 0.026). Similarly, the *D* allele was significantly associated with more severe disease presentation (OR =2.359; *P* = 0.014: Supplementary Table 3 and Figure 2).

### Hospitalization

Among the 232 cases, 144 (62.1%) were non-hospitalized while 88 (37.9%) were hospitalized. The mean ± SD of length of stay was 13.45 ± 13.73 days.

It is shown in Table 3 that hospitalized patients were older and of higher BMI. There was a significantly larger proportion of hospitalized males compared to non-hospitalized males. Dyslipidemia, hypertension, diabetes, heart disease, kidney disease, coagulation disorders, and cancer were significant comorbid predictors for hospitalization. Additionally, there was a larger proportion of ACEI/ARB_s_ intake among hospitalized cases (Table 3).

**Table 3:** Association between baseline characteristics and *ACE1* polymorphism with hospitalized vs. non-hospitalized COVID-19 cases.

			**Not hospitalized** ***N*** **= 144**	**Hospitalized** ***N*** **= 88**	***P*-Value^a^**
Age (years)	Mean ± SD		36.49 ± 11.28	55.64 ± 15.09	**<0.001**
BMI^b^ (kg/m^2^)	Mean ± SD		26.50 ± 4.81	29.98 ± 5.92	**<0.001**
Sex	Female	*N* (%)	79 (54.9)	27 (30.7)	**<0.001**
	Male	*N* (%)	65 (45.1)	61 (69.3)	
Blood group A+	Yes	*N* (%)	75 (52.1)	43 (48.9)	0.367
	No	*N* (%)	69 (47.9)	45 (51.1)	
Smoking	Ever	*N* (%)	62 (43.1)	36 (40.9)	0.428
	Never	*N* (%)	82 (56.9)	52 (59.1)	
Dyslipidemia	Yes	*N* (%)	15 (10.4)	25 (28.4)	<**0.001**
	No	*N* (%)	129 (89.6)	63 (71.6)	
Hypertension	Yes	*N* (%)	10 (6.9)	36 (40.9)	**<0.001**
	No	*N* (%)	134 (93.1)	52 (59.1)	
Diabetes	Yes	*N* (%)	4 (2.8)	25 (28.4)	**<0.001**
	No	*N* (%)	140 (97.2)	63 (71.6)	
Heart disease^c^	Yes	*N* (%)	1 (0.7)	14 (15.9)	**<0.001**
	No	*N* (%)	143 (99.3)	74 (84.1)	
Kidney disease^d^	Yes	*N* (%)	1 (0.7)	7 (8.0)	**0.005**
	No	*N* (%)	143 (99.3)	81 (92.0)	
Lung disease^e^	Yes	*N* (%)	7 (4.9)	6 (6.8)	0.362
	No	*N* (%)	137 (95.1)	82 (93.2)	
Cerebrovascular disease^f^	Yes	*N* (%)	0 (0.0)	2 (2.3)	0.143
	No	*N* (%)	144 (100)	86 (97.7)	
Coagulation disorders^g^	Yes	*N* (%)	0 (0.0)	4 (4.5)	**0.020**
	No	*N* (%)	144 (100)	84 (95.5)	
Cancer	Yes	*N* (%)	1 (0.7)	20 (22.7)	<**0.001**
	No	*N* (%)	143 (99.3)	64 (77.3)	
ACEI^h^/ARB^i^	Yes	*N* (%)	9 (6.3)	18 (20.5)	**0.001**
	No	*N* (%)	135 (93.8)	70 (79.5)	
*ACE* genotype	*II*	*N* (%)	22 (15.3)	11 (12.5)	0.555
	*DI*	*N* (%)	67 (46.5)	37 (42.0)	
	*DD*	*N* (%)	55 (38.2)	40 (45.5)	
	*II*	*N* (%)	22 (15.3)	11 (12.5)	0.351
	*DI* + *DD*	*N* (%)	122 (84.7)	77 (87.5)	
	*DI* + *II*	*N* (%)	89 (61.8)	48 (54.5)	0.170
	*DD*	*N* (%)	55 (38.2)	40 (45.5)	
*ACE* allele	*I*	*N* (%)	111 (38.5)	59 (33.5)	0.161
	*D*	*N* (%)	177 (61.5)	117 (66.5)	

a *P-values defined using independent t-test for continuous variables and Fisher exact for categorical variables*.

b *Body mass index*.

c *Coronary artery disease; heart failure*.

d *Chronic kidney disease, end-stage renal disease*.

e *Chronic obstructive pulmonary disease, interstitial lung disease, asthma*.

f *Stroke, carotid stenosis*.

g *Hemophilia, von Willebrand disease*.

h *Angiotensin converting enzyme inhibitor*.

i *Angiotensin receptor blocker*.

*The statistically significant P values are in bold*.

**Figure 3:**
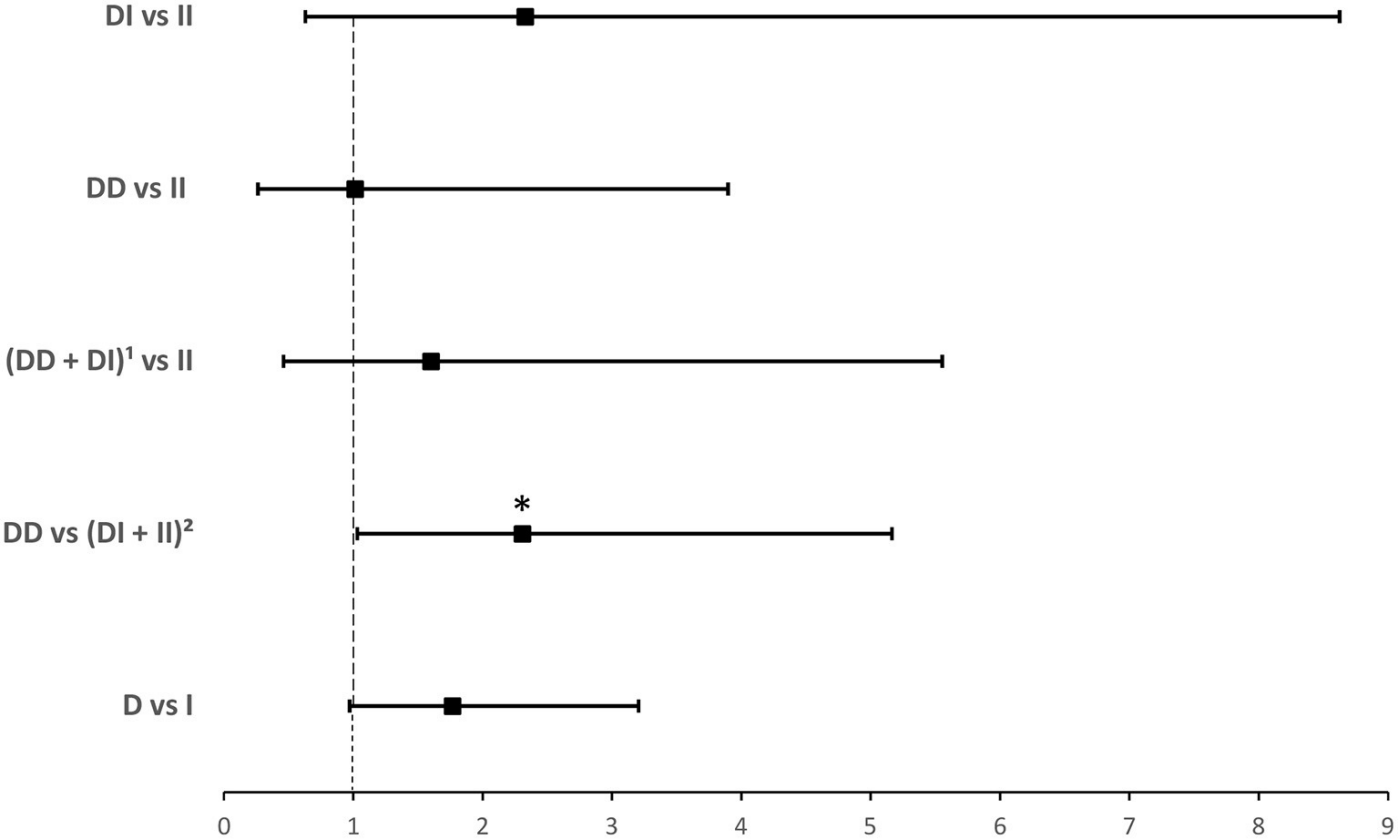
Forest plot showing adjusted odds ratios ± 95% confidence intervals of *ACE1* polymorphism for hospitalization for COVID-19. Multivariate analysis included variables that were statistically significant in the association analysis shown in Table 3; ^*^P < 0.05. ^1^*D* allele carriers, ^2^*I* allele carriers.

*ACE1 I/D* genotype and allele frequencies were not significantly associated with hospitalization although there was a trend of higher *DD* genotype and *D* allele frequencies in hospitalized cases with COVID-19 disease (Table 3). After adjusting for age, BMI, sex, significant comorbidities, and ACEI/ARB_s_ intake, binary logistic regression showed that, compared to *I* carriers, individuals with the *DD* genotype were at higher risk for hospitalization following infection (OR = 2.307; *P* = 0.042; Supplementary Table 4 and Figure 3).

### Hypoxia

Among the 88 hospitalized patients, 26 (29.5%) were not hypoxic as opposed to 62 (70.5%) that were.

As shown in Table 4, hypoxic patients only had significantly higher BMI when compared to admitted patients without hypoxia. There was a slightly larger proportion of hypoxic males compared to non-hypoxic males, but this result was not statistically significant. There were no significant comorbid predictors for developing hypoxia; nevertheless, there was an increasing trend for dyslipidemia, hypertension, diabetes, and ACEI/ARB_s_ intake among hypoxic patients (Table 4).

**Table 4:** Association between baseline characteristics and *ACE1* polymorphism with hypoxic vs. non-hypoxic hospitalized COVID-19 cases.

			**Not hypoxic** ***N*** **= 26**	**Hypoxic** ***N*** **= 62**	***P*** **-Value^a^**
Age (years)	Mean ± SD		52.08 ± 14.38	57.13 ± 15.24	0.146
BMI^b^ (kg/m^2^)	Mean ± SD		27.27 ± 4.35	31.12 ± 6.15	**0.001**
Sex	Female	*N* (%)	9 (34.6)	18 (29.0)	0.391
	Male	*N* (%)	17 (65.4)	44 (71.0)	
Blood group A+	Yes	*N* (%)	11 (42.3)	32 (51.6)	0.287
	No	*N* (%)	15 (57.7)	30 (48.4)	
Smoking	Ever	*N* (%)	11 (42.3)	25 (40.3)	0.523
	Never	*N* (%)	15 (57.7)	37 (59.7)	
Dyslipidemia	Yes	*N* (%)	4 (15.4)	21 (33.9)	0.064
	No	*N* (%)	22 (84.6)	41 (66.1)	
Hypertension	Yes	*N* (%)	7 (26.9)	29 (46.8)	0.067
	No	*N* (%)	19 (73.1)	33 (53.2)	
Diabetes	Yes	*N* (%)	6 (23.1)	19 (30.6)	0.328
	No	*N* (%)	20 (76.9)	43 (69.4)	
Heart disease^c^	Yes	*N* (%)	5 (15.4)	10 (16.1)	0.603
	No	*N* (%)	22 (84.6)	52 (83.9)	
Kidney disease^d^	Yes	*N* (%)	3 (11.5)	4 (6.5)	0.339
	No	*N* (%)	23 (88.5)	58 (93.5)	
Lung disease^e^	Yes	*N* (%)	3 (11.5)	3 (4.8)	0.242
	No	*N* (%)	23 (88.5)	59 (95.2)	
Cerebrovascular disease^f^	Yes	*N* (%)	0 (0.0)	2 (3.2)	0.494
	No	*N* (%)	26 (100.0)	60 (96.8)	
Coagulation disorders^g^	Yes	*N* (%)	0 (0.0)	4 (6.5)	0.239
	No	*N* (%)	26 (100.0)	58 (93.5)	
Cancer	Yes	*N* (%)	9 (34.6)	11 (17.7)	0.077
	No	*N* (%)	17 (65.4)	51 (82.3)	
ACEI^h^/ARB^i^	Yes	*N* (%)	3 (11.5)	15 (24.2)	0.145
	No	*N* (%)	23 (88.5)	47 (75.8)	
*ACE* genotype	*II*	*N* (%)	6 (23.1)	5 (8.1)	0.171
	*DI*	*N* (%)	10 (38.5)	27 (43.5)	
	*DD*	*N* (%)	10 (38.5)	30 (48.4)	
	*II*	*N* (%)	6 (23.1)	5 (8.1)	0.060
	*DI* + *DD*	*N* (%)	20 (76.9)	57 (91.9)	
	*DI* + *II*	*N* (%)	16 (61.5)	32 (51.6)	0.269
	*DD*	*N* (%)	10 (38.5)	30 (48.4)	
*ACE* allele	*I*	*N* (%)	22 (42.3)	37 (29.8)	0.078
	*D*	*N* (%)	30 (57.7)	87 (70.2)	

a *P-values defined using independent t-test for continuous variables and Fisher exact for categorical variables*.

b *Body mass index*.

c *Coronary artery disease; heart failure*.

d *Chronic kidney disease, end-stage renal disease*.

e *Chronic obstructive pulmonary disease, interstitial lung disease, asthma*.

f *Stroke, carotid stenosis*.

g *Hemophilia, von Willebrand disease*.

h *Angiotensin converting enzyme inhibitor*.

i *Angiotensin receptor blocker*.

*The statistically significant P values are in bold*.

**Figure 4:**
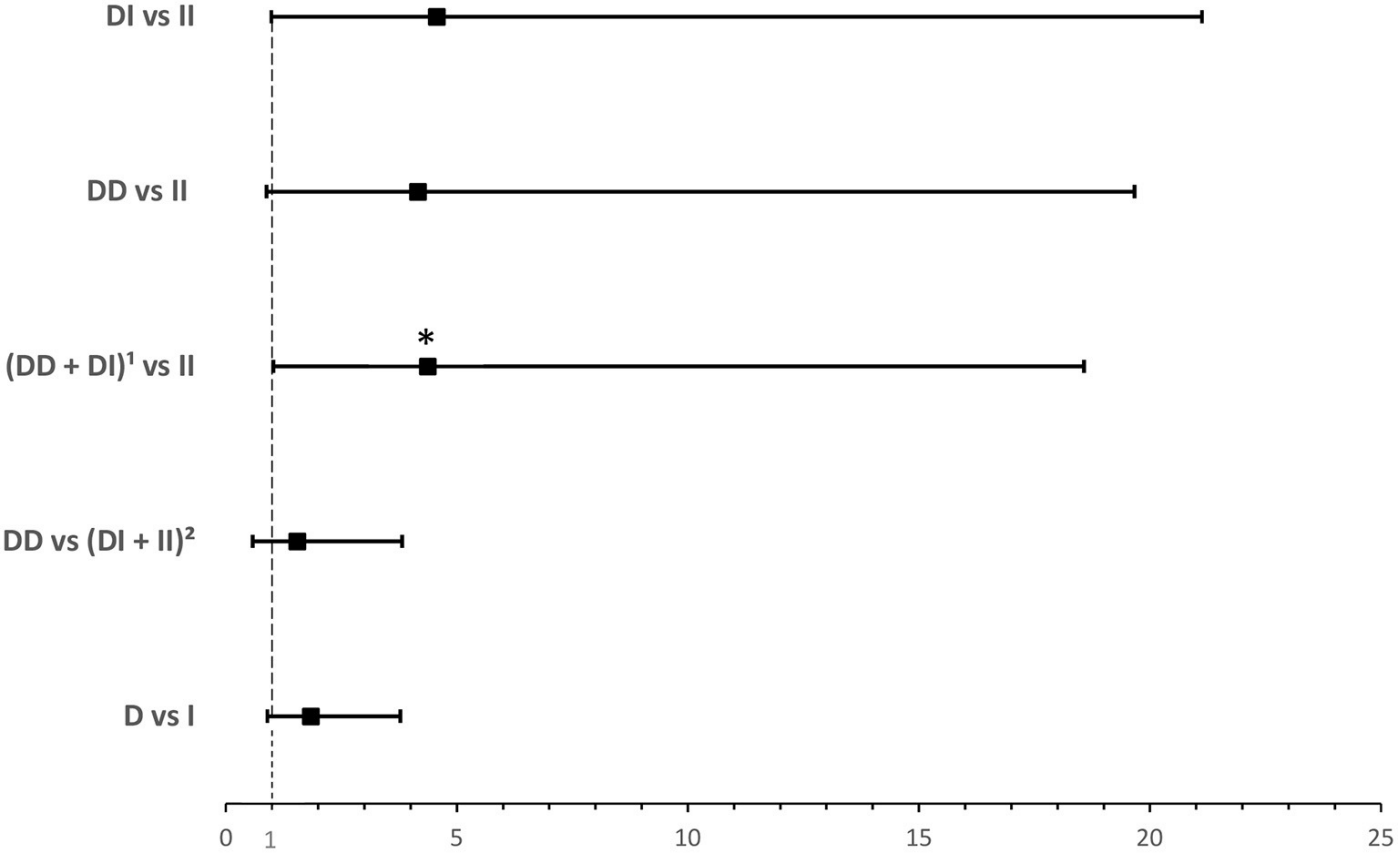
Forest plot showing adjusted odds ratios ± 95% confidence intervals of *ACE1* polymorphism for developing hypoxia in hospitalized COVID-19 cases. Multivariate analysis adjusted for BMI; ^*^P < 0.05. ^1^*D* allele carriers. ^2^*I* allele carriers.

*ACE1 I/D* genotype and allele frequencies were not significantly associated with hypoxia although there was a trend of higher *DD* genotype and *D* allele frequencies in hypoxic hospitalized cases with COVID-19 disease (Table 4). After adjusting for BMI, binary logistic regression showed that, compared to the *II* genotype, *D* allele carriers were at an increased risk for developing hypoxia following infection (OR = 4.374; *P* = 0.045; Supplementary Table 5 and Figure 4).

## Discussion

Ever since the outbreak, people realized that the SARS-CoV-2 virus hits every individual differently with varying symptoms and severity. There has been a plethora of articles from different populations and ethnicities discussing the factors that are considered to be risk factors for both symptoms and severity of the COVID-19 disease, but with only few related to genetics. This study is the first to evaluate these factors in Lebanese Arabs. We show that almost all previously reported factors and comorbidities also predict disease susceptibility and severity in the Lebanese population. We also show a positive correlation between *ACE1 I* and the risk of contracting the COVID-19 disease, and between *ACE1 D* and worse COVID-19 infection. These results suggest that genotyping for *ACE1 I/D* polymorphism could be used to assess risk and predict severity for better prognosis and management of the disease. This is especially important for Middle Easterners in general and the Lebanese in particular who, and similarly to the results of the current study, have a higher frequency of the *ACE1 D* allele when compared to the *I* allele (24, 25).

### Demographics, Health Related Behaviors, and Comorbidities

Most of the associated demographics, health-related behaviors, and comorbidities can be explained at the physiological level. For instance for age, *ACE2* receptor, being the key factor in the entry of the virus, is more highly expressed in well-differentiated ciliated epithelial cells found in adults (2). Moreover, the immunity of an older individual is weaker than the immunity of children due to immunosenescence and the presence of central memory T cells rather than naïve T cells (2). Our results agree with the literature since the mean age (in years) is significantly higher in the infected cases when compared to the non-infected controls, and it is significantly higher with disease severity. Concerning sex, *ACE2* being an X-linked gene can be considered as a disadvantage for infected males, since lower *ACE2* expression may correlate with lesser conversion of Angiotensin II into Angiotensin 1–7 (28). Moreover, testosterone suppresses the immune system in males, which affects the T cell responses (29). These findings are compatible with our results that show that the majority of cases and those with worse outcome are males. In our study, the mean BMI (kg/m^2^) was also significantly higher in the infected cases and associated with more severe disease. This can be explained by the fact that the adipose tissue expresses *ACE2* receptors as much as the pulmonary tissues (7). Accordingly, obese individuals have higher levels of circulating *ACE2* with secondarily higher disease susceptibility and adverse outcome (30). As for blood group, data are still non-conclusive. For example, it has been shown that carriage of blood group A was associated with a higher rate of COVID-19 infection when compared to blood group O (31). However, it is felt that individuals with blood group A also have more underlying comorbidities (29), which could be the reason behind the significance seen in infected patients. In our study, blood group did not show any significant difference with neither risk nor severity of the disease.

Concerning health-related behavior, smoking is one of the most common risk factor for many diseases. That is why smoking is expected to further complicate the symptoms of COVID-19. Smoking is shown to increase the gene expression of *ACE2* in the lungs (4). Moreover, nicotine upregulates the activity of renin and *ACE1* thus activating ACE/Angiotensin II/AT1R pathway, and decreases the activity of AT2R by downregulating the activity of *ACE2* (32). A systematic review has shown that current smokers had a lower risk for developing severe outcome when compared to former smokers (33). However, a preliminary meta-analysis on five studies in China, and similarly to our results, has shown that active smoking is not significantly related to the severity of COVID-19 (34). Further data are needed to resolve this controversy.

To date, it is still unclear whether ACEI and/or ARB_s_ should be kept in patients who contract COVID-19. There are currently two contradicting hypotheses in the literature that RAS inhibition could be both harmful and protective (8). In our study, ACEI/ARB_s_ were significantly more frequently taken in the worse disease outcome group. However, it is possible that these results relate to the fact that this group of subjects has underlying comorbidities that necessitate ACEI/ARB_s_ treatment. As a matter of fact, people with underlying comorbidities such as diabetes, hypertension (HTN), cardiovascular diseases (CVD), chronic kidney diseases (CKD), lung diseases (COPD and asthma), cerebral vascular disease, and coagulation disorders are at a higher risk of worse COVID-19 severity and outcome (35–37). Our results clearly show that comorbidities are risk factors for contracting the virus and developing a worse COVID-19 disease outcome.

### *ACE1* I/D Genetic Polymorphism

In relation to disease susceptibility, available data, most of which are literature and database searches, are at times contradictory (21). For example on one hand, Yamamoto et al. (23) showed that countries with higher frequency of the *ACE1 I* allele had less susceptibility to COVID-19. On the other hand, Delanghe et al. (22) showed that a high frequency of *ACE1 I* allele increases the prevalence of COVID-19 cases. Nevertheless, when Yamamoto et al. (23) specifically looked at Middle Eastern populations, they found a weaker association with the *D* allele, hence the need for further investigations. To our knowledge, we are the first to evaluate such an association in patients. We confirmed Delanghe et al.'s (22) simulations by showing that the frequency of *II* was significantly highest in infected cases when compared to controls coupled with a significantly higher risk of contracting the COVID-19 disease after adjusting for confounders.

As for disease outcome, *ACE1 DD* genotype leads to higher activity of *ACE1* enzyme thus lowering *ACE2* causing an increase in the amount of angiotensin II left active. Although lower levels of *ACE2* could mean that there is less chance for SARS-CoV-2 to bind and enter the host cell, high levels of angiotensin II would act through AT1R and further cause cardiovascular and lung pathologies (16). As a matter of fact, Gomez et al. (16) found that *ACE1 DD* genotype was more frequent in severe COVID-19 cases, suggesting that there is an association between *ACE1 DD* genotype and the severity of COVID-19. Furthermore, *ACE1 DD* genotype has been correlated with respiratory failure (12) and increased death rate (38) in patients infected with COVID-19. In addition, an ecologic meta-regression showed that there is a link between *ACE1 I/D* polymorphism and the recovery rate of COVID-19 whereby faster recovery was correlated with higher frequency ratio of the *I/D* allele (20). Our results are in agreement with the literature. Notably, it could be argued that the latter association is due to the known associations of the *ACE1 D* allele with cardiovascular comorbidities. In our cohort of infected cases however, we found no significant associations with any of the comorbidities (Supplementary Table 6).

### Limitations

This study has few limitations. First, the sample size is limited to a single country and institution, and is relatively small. Of note that we did not estimate needed sample size at study initiation because of lack of such data at the time and the study being exploratory. Nevertheless, our sample size for the severity outcome is very similar to two recent investigations, one with Spanish Caucasians (16) and another with Indians (39). Second, the study entailed multiple testing, the adjustment of which could lead to loss of statistical significance. In fact for the severity outcome whereby we assessed three independent outcomes, it may be relevant to set the significance level at 0.016 (0.05/3). With such adjustment, only the association between the *D* allele and disease severity remains statistically significant (OR =2.359; *P* = 0.014). Notably, disease severity was classified as mild, moderate, and severe as per the WHO progression scale scoring system (26), a scoring system that is based on a constellation of assessment tools for severity following infection that includes hospitalization status, oxygen saturation, and need for oxygen therapy. With a larger representative sample, it is possible to have independently increased risks for both hospitalization and hypoxia with the *D* allele after accounting for multiple testing (*P* < 0.016). Additional data from other institutions and populations may address these two limitations with the opportunity to perform a meta-analysis. Third, the study only evaluated the *ACE1 I/D* polymorphism and did not look at other possible SNPs in *ACE1*. Moreover, it would be relevant to look at *ACE2* and *TMPRSS2* variants, as these two genes are important factors in the entry of SARS-CoV-2 (40). Finally, the role of ACEI/ARB_s_ in COVID-19 disease is still unresolved and it would be interesting to evaluate whether there is any interaction between *ACE* polymorphisms and these drugs in the SARS-COV2 setting (41).

## Conclusion

To our knowledge, we are the first to evaluate the association of *ACE1* genetic polymorphism with COVID-19 disease susceptibility and outcome in a Middle Eastern Arab population such as the Lebanese. Despite its limitations, results of this study suggest that genotyping for *ACE1 I/D* polymorphism could be used to elicit the disease risk and severity for better prognosis and management. Further studies are needed to evaluate additional genetic variants in different ethnicities and populations.

## Data Availability Statement

The original contributions presented in the study are included in the article/Supplementary Material, further inquiries can be directed to the corresponding author/s.

## Ethics Statement

The studies involving human participants were reviewed and approved by the American University of Beirut Institutional Review Board under protocol: BIO-2020-0259. The patients/participants provided their written informed consent to participate in this study.

## Author Contributions

CS, RM, IA, and NZ contributed conception and design of the study. HS recruited study subjects and collected data. KJ performed the experiments. HS, KJ, and NZ organized the database, performed the statistical analysis, and wrote the first draft of the manuscript. All authors contributed to manuscript revision, read, and approved the submitted version.

## Funding

This work was supported by National Council for Scientific Research Lebanon: The flash call COVID-19 management in Lebanon. Diana Tamari Sabbagh Scholars Program (DTSSP)—Award for MS Graduate Students in biomedical research.

## Conflict of Interest

The authors declare that the research was conducted in the absence of any commercial or financial relationships that could be construed as a potential conflict of interest.

## Publisher's Note

All claims expressed in this article are solely those of the authors and do not necessarily represent those of their affiliated organizations, or those of the publisher, the editors and the reviewers. Any product that may be evaluated in this article, or claim that may be made by its manufacturer, is not guaranteed or endorsed by the publisher.
