# Supplementary material for: The Role of Angiotensin Converting Enzyme 1 Insertion/Deletion Genetic Polymorphism in the Risk and Severity of COVID-19 Infection
Source: Front Med (Lausanne). 2021 Dec 23;8:798571. doi: 10.3389/fmed.2021.798571 (PMC8733297; doi:10.3389/fmed.2021.798571)
Supplement: Supplementary file 1 [file Table_1.docx]

**The Role of Angiotensin Converting Enzyme 1 (ACE1) Insertion/Deletion Genetic Polymorphism in the Risk and Severity of COVID-19 infection**

**Supplementary Table 1.** Frequency of the *ACE II* (rs1799752) genotype in different ethnicities and geographic areas (24;27)

| **Ethnicity or geographic area** | ***ACE II* Frequency (%)** |
| --- | --- |
| USA-Caucasians | 18 |
| USA-African Americans | 17 |
| Europe | 10-27¹ |
| Middle East | 2-24¹ |
| Lebanese (current study) | 7.8 |
| Korea | 34-37^2^ |
| Japan | 41-51^2^ |
| Taiwan | 47-50^2^ |
| China | 33-59^2^ |

1. Range depending on country
2. Range depending on source
