# Supplementary material for: The Role of Angiotensin Converting Enzyme 1 Insertion/Deletion Genetic Polymorphism in the Risk and Severity of COVID-19 Infection
Source: Front Med (Lausanne). 2021 Dec 23;8:798571. doi: 10.3389/fmed.2021.798571 (PMC8733297; doi:10.3389/fmed.2021.798571)
Supplement: Supplementary file 2 [file Table_2.docx]

**Supplementary Table 2.** Association between *ACE1* polymorphism and COVID-19 positive cases vs. COVID-19 negative controls

|  | |  | | **OR** | **95% CI** | **P-Value^1^** |
| --- | --- | --- | --- | --- | --- | --- |
| ***ACE1* GENOTYPE** | | | | | |  |
| ***II & DI vs DD*** | **Univariate** | | **DD** | **Ref ^2^** | - | - |
|  |  |  | **DI** | 1.080 | 0.702-1.659 | 0.727 |
|  |  |  | **II** | 2.055 | 0.992-4.259 | 0.053 |
|  | **Multivariate** | | **DD** | **Ref^2^** | - | - |
|  |  |  | **DI** | 1.012 | 0.642-1.596 | 0.959 |
|  |  |  | **II** | 2.086 | 0.978-4.451 | 0.057 |
| ***II vs (DD+DI)^3^*** | **Univariate** | | **DD+DI** | **Ref^2^** | - | - |
|  |  |  | **II** | 1.976 | 0.986-3.959 | 0.055 |
|  | **Multivariate** | | **DD+DI** | **Ref^2^** | - | - |
|  |  |  | **II** | **2.074** | **1.005 – 4.280** | **0.048** |
| ***(DI+II)^4^ vs DD*** | **Univariate** | | **DD** | **Ref^2^** | - | - |
|  |  |  | **DI+II** | 1.219 | 0.809-1.837 | 0.344 |
|  | **Multivariate** | | **DD** | **Ref^2^** | - | - |
|  |  |  | **DI+II** | 1.168 | 0.759 – 1.800 | 0.480 |
| ***ACE1* ALLELE** | | | | | | |
| ***I vs D*** | **Univariate** | | **D** | **Ref^2^** | - | - |
|  |  |  | **I** | 1.289 | 0.949 – 1.750 | 0.104 |
|  | **Multivariate** | | **D** | **Ref^2^** | - | - |
|  |  |  | **I** | 1.272 | 0.923 – 1.754 | 0.142 |

1. P-value defined using binary logistic regression with Odds Ratio (OR) and 95% Confidence Interval (CI). Multivariate analysis included variables that were statistically significant in the association analysis shown in Table 1. Statistically significant results are in bold.
2. The Genotype/combination used as reference
3. *D-*carriers
4. *I-*carriers
