# Supplementary material for: The Role of Angiotensin Converting Enzyme 1 Insertion/Deletion Genetic Polymorphism in the Risk and Severity of COVID-19 Infection
Source: Front Med (Lausanne). 2021 Dec 23;8:798571. doi: 10.3389/fmed.2021.798571 (PMC8733297; doi:10.3389/fmed.2021.798571)
Supplement: Supplementary file 3 [file Table_3.docx]

**Supplementary Table 3.** Association between baseline characteristics and *ACE1* polymorphism with disease severity^1^ in symptomatic COVID-19 cases

|  | | | **Moderate** | | | **Severe** | | |
| --- | --- | --- | --- | --- | --- | --- | --- | --- |
|  |  |  | **OR** | **95% CI** | **P-Value^2^** | **OR** | **95% CI** | **P-Value^2^** |
| ***ACE1* GENOTYPE** | | | | | | | |  |
| ***DD & DI vs II*** | **Univariate** | **II** | **Ref ^3^** | - | - | **Ref ^3^** | - | - |
|  |  | **DI** | 0.890 | 0.283 – 2.806 | 0.843 | 1.929 | 0.662 – 5.624 | 0.229 |
|  |  | **DD** | 0.812 | 0.245 – 2.692 | 0.733 | 2.706 | 0.931 – 7.866 | 0.067 |
|  | **Multivariate** | **II** | **Ref^3^** | - | - | **Ref ^3^** | - | - |
|  |  | **DI** | 1.482 | 0.266 – 8.250 | 0.654 | 2.438 | 0.455 – 13.053 | 0.298 |
|  |  | **DD** | 2.247 | 0.407 – 12.411 | 0.353 | **5.751** | **1.105 – 29.901** | **0.038** |
| ***(DD*+*DI)^4^ vs II*** | **Univariate** | **II** | **Ref^3^** | - | - | **Ref ^3^** | - | - |
|  |  | **DD+DI** | 0.855 | 0.292 – 2.501 | 0.775 | 2.800 | 0.823 – 6.314 | 0.113 |
|  | **Multivariate** | **II** | **Ref^3^** | - | - | **Ref ^3^** | - | - |
|  |  | **DD+DI** | 1.859 | 0.371 – 9.323 | 0.451 | 3.916 | 0.808 – 18.987 | 0.090 |
| ***DD vs (DI+II)^5^*** | **Univariate** | **DI+II** | **Ref^3^** | - | - | **Ref ^3^** | - | - |
|  |  | **DD** | 0.882 | 0.366 – 2.126 | 0.780 | 1.613 | 0.876 – 2.969 | 0.125 |
|  | **Multivariate** | **DI+II** | **Ref^3^** | - | - | **Ref ^3^** | - | - |
|  |  | **DD** | 1.644 | 0.561 – 4.822 | 0.365 | **2.845** | **1.133 – 7.142** | **0.026** |
| ***ACE1* ALLELE** | | | | | | | | |
| ***D vs I*** | **Univariate** | **I** | **Ref^3^** | - | - | **Ref^3^** | - | - |
|  |  | **D** | 0.898 | 0.492 – 1.639 | 0.726 | 1.573 | 0.994 – 2.489 | 0.053 |
|  | **Multivariate** | **I** | **Ref^3^** | - | - | **Ref^3^** | - | - |
|  |  | **D** | 1.508 | 0.694 – 3.278 | 0.300 | **2.359** | **1.188 – 4.682** | **0.014** |

1. Rated as mild, moderate, or severe according to WHO clinical progression scale for COVID-19 with mild as Reference.
2. P-value defined using multinomial logistic regression with Odds Ratio (OR) and 95% Confidence Interval (CI). Multivariate analysis included variables that were statistically significant in the association analysis shown in Table 2. Statistically significant results are in bold.
3. The Genotype/combination used as reference
4. *D-*carriers
5. *I-*carriers
