# Supplementary material for: The Role of Angiotensin Converting Enzyme 1 Insertion/Deletion Genetic Polymorphism in the Risk and Severity of COVID-19 Infection
Source: Front Med (Lausanne). 2021 Dec 23;8:798571. doi: 10.3389/fmed.2021.798571 (PMC8733297; doi:10.3389/fmed.2021.798571)
Supplement: Supplementary file 4 [file Table_4.docx]

**Supplementary Table 4.** Association between baseline characteristics and *ACE1* polymorphism with non-hospitalized vs hospitalized COVID-19 cases.

|  | | | **OR** | **95% CI** | **P-Value^1^** |
| --- | --- | --- | --- | --- | --- |
| ***ACE1* GENOTYPE** | | | | |  |
| ***DD & DI vs II*** | **Univariate** | **II** | **Ref ^2^** | - | - |
|  |  | **DI** | 1.455 | 0.634 – 3.337 | 0.377 |
|  |  | **DD** | 1.104 | 0.483 – 2.527 | 0.814 |
|  | **Multivariate** | **II** | **Ref ^2^** | - | - |
|  |  | **DI** | 2.329 | 0.629 -8.625 | 0.205 |
|  |  | **DD** | 1.013 | 0.263-3.897 | 0.985 |
| ***(DI*+*DD)^3^ vs II*** | **Univariate** | **II** | **Ref ^2^** | - | - |
|  |  | **DD+DI** | 1.262 | 0.580 – 2.748 | 0.557 |
|  | **Multivariate** | **II** | **Ref ^2^** | - | - |
|  |  | **DD+DI** | 1.600 | 0.461 – 5.553 | 0.459 |
| ***DD vs (DI+ II)^4^*** | **Univariate** | **DI+II** | **Ref ^2^** | - | - |
|  |  | **DD** | 1.348 | 0.788 – 2.309 | 0.276 |
|  | **Multivariate** | **DI+II** | **Ref ^2^** | - | - |
|  |  | **DD** | **2.307** | **1.030 – 5.165** | **0.042** |
| ***ACE1* ALLELE** | | | | | |
| ***D vs I*** | **Univariate** | **I** | **Ref ^2^** | - | - |
|  |  | **D** | 1.244 | 0.840 – 1.842 | 0.277 |
|  | **Multivariate** | **I** | **Ref ^2^** | - | - |
|  |  | **D** | 1.765 | 0.972 – 3.205 | 0.062 |

1. P-value defined using binary logistic regression Odds Ratio (OR) and 95% Confidence Interval (CI). Multivariate analysis included variables that were statistically significant in the association analysis shown in Table 3. Statistically significant results are in bold.
2. The Genotype/combination used as reference
3. *D-*carriers
4. *I-*carriers
