# Supplementary material for: The Role of Angiotensin Converting Enzyme 1 Insertion/Deletion Genetic Polymorphism in the Risk and Severity of COVID-19 Infection
Source: Front Med (Lausanne). 2021 Dec 23;8:798571. doi: 10.3389/fmed.2021.798571 (PMC8733297; doi:10.3389/fmed.2021.798571)
Supplement: Supplementary file 5 [file Table_5.docx]

**Supplementary Table 5**. Association between baseline characteristics and *ACE1* polymorphism with non-hypoxic vs hypoxic hospitalized COVID-19 cases

|  | | | **OR** | **95% CI** | **P-Value^1^** |
| --- | --- | --- | --- | --- | --- |
| ***ACE1* GENOTYPE** | | | | | |
| ***DD & DI vs II*** | **Univariate** | **II** | **Ref ^2^** | - | - |
|  |  | **DI** | 3.600 | 0.900 –14.394 | 0.070 |
|  |  | **DD** | 3.240 | 0.806 –13.021 | 0.098 |
|  | **Multivariate** | **II** | **Ref^2^** | - | - |
|  |  | **DI** | 4.568 | 0.988 –21.126 | 0.052 |
|  |  | **DD** | 4.162 | 0.881 –19.667 | 0.072 |
| ***(DD*+*DI)^3^ vs II*** | **Univariate** | **II** | **Ref^2^** | - | - |
|  |  | **DD+DI** | 3.420 | 0.940 – 12.443 | 0.062 |
|  | **Multivariate** | **II** | **Ref^2^** | - | - |
|  |  | **DD+DI** | **4.374** | **1.030 – 18.572** | **0.045** |
| ***DD vs (DI+II)^4^*** | **Univariate** | **DI+II** | **Ref^2^** | - | - |
|  |  | **DD** | 1.500 | 0.589 – 3.817 | 0.395 |
|  | **Multivariate** | **DI+II** | **Ref^2^** | - | - |
|  |  | **DD** | 1.553 | 0.581 – 4.149 | 0.380 |
| ***ACE1* ALLELE** | | | | | |
| ***D vs I*** | **Univariate** | **I** | **Ref^2^** | - | - |
|  |  | **D** | 1.724 | 0.881 – 3.374 | 0.112 |
|  | **Multivariate** | **I** | **Ref^2^** | - | - |
|  |  | **D** | 1.846 | 0.902 – 3.778 | 0.094 |

1. P-value defined using binary logistic regression Odds Ratio (OR) and 95% Confidence Interval (CI). Multivariate analysis included variables that were statistically significant in the association analysis shown in Table 4. Statistically significant results are in bold.
2. The Genotype/combination used as reference
3. *D-*carriers
4. *I-*carriers
