# Supplementary material for: The Role of Angiotensin Converting Enzyme 1 Insertion/Deletion Genetic Polymorphism in the Risk and Severity of COVID-19 Infection
Source: Front Med (Lausanne). 2021 Dec 23;8:798571. doi: 10.3389/fmed.2021.798571 (PMC8733297; doi:10.3389/fmed.2021.798571)
Supplement: Supplementary file 6 [file Table_6.docx]

**Supplementary Table 6.** Association between *ACE1* polymorphism and comorbidities in COVID-19 positive cases

|  | | | | | | ***ACE1 Genotype*** | | | ***ACE1***  ***Allele*** |
| --- | --- | --- | --- | --- | --- | --- | --- | --- | --- |
|  | | | | | | ***II vs DI vs DD*** | ***(DI+DD)^1^*** | ***(DI+II)^2^*** | ***D vs I*** |
|  | | | ***II***  ***N=33*** | ***DI***  ***N=104*** | ***DD***  ***N=95*** | **P-value^3^** | **P-value^3^** | **P-value^3^** | **P-value^3^** |
| **Dyslipidemia** | **Yes** | N (%) | 5 (15.2) | 21 (20.2) | 14 (14.7) | 0.589 | 0.255 | 0.478 | 0.378 |
|  | **No** | N (%) | 28 (84.8) | 83 (79.8) | 81 (85.3) |  |  |  |  |
| **Hypertension** | **Yes** | N (%) | 5 (15.2) | 25 (24.0) | 16 (16.8) | 0.355 | 0.218 | 0.321 | 0.422 |
|  | **No** | N (%) | 28 (84.8) | 79 (76.0) | 79 (83.2) |  |  |  |  |
| **Diabetes** | **Yes** | N (%) | 1 (3.0) | 17 (16.3) | 11 (11.6) | 0.127 | 0.444 | 0.056 | 0.308 |
|  | **No** | N (%) | 32 (97.0) | 87 (83.7) | 84 (88.4) |  |  |  |  |
| **Heart Disease^4^** | **Yes** | N (%) | 1 (3.0) | 10 (9.6) | 4 (4.2) | 0.252 | 0.188 | 0.340 | 0.415 |
|  | **No** | N (%) | 32 (97.0) | 94 (90.4) | 91 (95.8) |  |  |  |  |
| **Kidney Disease^5^** | **Yes** | N (%) | 1 (3.0) | 5 (4.8) | 2 (2.1) | 0.703 | 0.292 | 0.682 | 0.361 |
|  | **No** | N (%) | 32 (97.0) | 99 (95.2) | 93 (97.9) |  |  |  |  |
| **Lung Disease^6^** | **Yes** | N (%) | 2 (6.1) | 5 (4.8) | 6 (6.3) | 0.860 | 0.453 | 0.577 | 0.502 |
|  | **No** | N (%) | 31 (93.9) | 99 (95.2) | 89 (93.7) |  |  |  |  |
| **Cerebrovascular Disease^7^** | **Yes** | N (%) | 0 (0.0) | 2 (1.9) | 0 (0.0) | 0.631 | 0.348 | 0.735 | 0.466 |
|  | **No** | N (%) | 95 (100.0) | 102 (98.1) | 33 (100.0) |  |  |  |  |
| **Coagulation Disorders^8^** | **Yes** | N (%) | 0 (0.0) | 2 (1.9) | 2 (2.1) | 1.000 | 0.542 | 0.539 | 0.388 |
|  | **No** | N (%) | 33 (100.0) | 102 (98.1) | 1. 97.9) |  |  |  |  |

1. *D-*carriers
2. *I-*carriers
3. P-value defined using Fisher exact test
4. Coronary artery disease; heart failure
5. Chronic kidney disease, end-stage renal disease
6. Chronic obstructive pulmonary disease, interstitial lung disease, asthma
7. Stroke, carotid stenosis
8. Hemophilia, von Willebrand disease
